# Improved efficiency for respiratory motion compensation in three-dimensional flow measurements

**DOI:** 10.1186/1532-429X-15-S1-P30

**Published:** 2013-01-30

**Authors:** Mehmet Akcakaya, Praveen Gulaka, Tamer A Basha, Thomas H Hauser, Warren J Manning, Reza Nezafat

**Affiliations:** 1 Medicine, Beth Israel Deaconess Medical Center, Harvard Medical School, Boston, MA, USA; 2 Radiology, Beth Israel Deaconess Medical Center, Harvard Medical School, Boston, MA, USA; 3 Health and Medical Equipment, Samsung Electronics Co., Suwon, Republic of Korea

## Background

Phase contrast (PC) CMR is clinically used for in-vivo assessment of blood flow in cardiovascular disease [1]. Typically, a through-plane 2D acquisition is performed for evaluating the blood flow. Recently, 3D time-resolved PC CMR has been used for quantification and visualization of the blood flow in all three directions of a volume [2]. However, such acquisitions require long scan times, which are further prolonged by the need for respiratory motion compensation, typically using respiratory navigators. In this study, we hypothesized that respiratory gating the center of k-space only will yield similar measurements to a fully respiratory-gated acquisition, since the phase information is mainly coming from the central k-space, and evaluated these two gating approaches.

## Methods

12 subjects were recruited (33.2±15.8 years; 5 males) for 3D flow CMR on a 1.5T Philips Achieva magnet. Images were acquired axially using a GRE sequence (TR/TE/α=5.7/3.4 ms/10^o^, resolution=2×2×4 mm^3^, FOV=340×340×40 mm^3^) in a volume covering the ascending and descending aorta, and the aortic bifurcation. Only foot-head flow encoding was used to provide an adequate temporal resolution of 23.2ms for the measurements. The nominal scan time for this scan was 12 minutes at 60 bpm.

For respiratory motion compensation, two gating & tracking strategies were used with a 7mm gating window: 1) All of k-space is acquired within the gating window (fully-gated), 2) Central k-space (corresponding to 4% of k-space) is acquired within the gating window, and the rest of k-space is acquired without any gating (center-gated). Two fully-gated acquisitions were performed. Acquisition time was recorded for each scan. Stroke volume measurements were performed both on the ascending and the descending aorta for all acquisitions. Bland-Altman analyses were performed to compare the flow measurements between different gating strategies (fully- vs. center-gated), as well as to compare the inter-scan variation for the fully-gated strategy (i.e., fully-gated #1 vs. #2).

## Results

**Figure 1:**
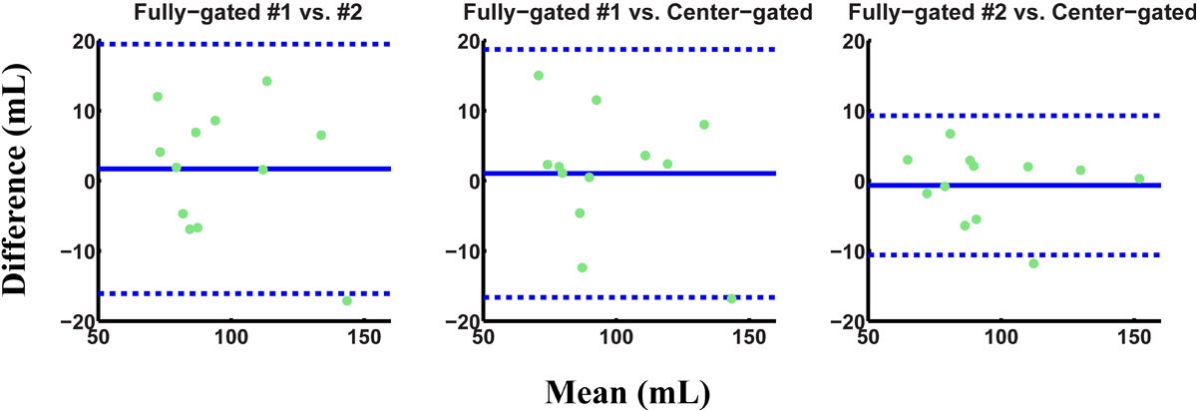
Bland-Altman analysis of the stroke volume through the ascending aorta for various navigator gating strategies. The center-gated scans are clinically identical to the fully-gated scans for these acquisitions.

**Figure 2:**
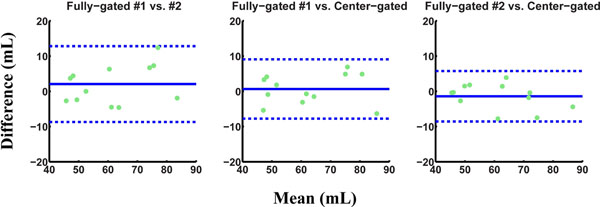
Bland-Altman analysis of the stroke volume through the descending aorta for fully-gated and center-gated strategies. The variability between fully-gated and center-gated scans is less than the inter-scan variability of fully-gated scans.

Figures 1 and 2 show the Bland-Altman analysis for stroke volume for the ascending and descending aorta respectively. The intra-scan variability observed for fully-gated scans is equivalent to or higher than the variability observed between fully-gated and center-gated strategies. Furthermore, the proposed center-gated strategy has significantly shorter acquisition time compared to the fully-gated strategy (13:19±3:02 vs. 19:35±5:02, P<0.001).

## Conclusions

We proposed and demonstrated a more efficient respiratory gating strategy for 3D PC CMR. No systematic variation was observed for the stroke volume (cardiac output) measurements between the proposed strategy and the fully-gated one, with the proposed strategy having a markedly shorter acquisition time.

## Funding

NIH: K99HL111410-01; R01EB008743-01A2; Samsung Electronics
